# Enhancing A82846B production by artificial *attB*-assisted overexpression of *orf10*–*orf11* genes in *Kibdelosporangium aridum* SIPI-3927

**DOI:** 10.1186/s13568-020-00992-x

**Published:** 2020-03-16

**Authors:** Xun Tian, He Huang, Hai-Feng Hu

**Affiliations:** 1 Shanghai Institute of Pharmaceutical Industry, China State Institute of Pharmaceutical Industry, Gebaini Road 285, Shanghai, 201203 China; 2 Sinopharm Health Industry Institute Co., Ltd., Gebaini Road 285, Shanghai, 201203 China; 3 Institute of Plant Physiology and Ecology Shanghai Institutes for Biological Sciences, Fenglin Road 300, Shanghai, 200032 China

**Keywords:** A82846B, *Kibdelosporangium aridum*, Halogenase, Glycosyltransferase, *attB*

## Abstract

A82846B, producing by *Kibdelosporangium aridum*, is an important precursor of the semi-synthetic glycopeptide antibiotic Oritavancin. *K. aridum* produces three components A82846A, B and C, so it is essential to increase A82846B titer and reduce A82846A and C titers by overexpressing halogenase and glycosyltransferase genes. Firstly, we constructed the genetically engineered strain SIPI-3927-*attB* harboring artificial *attB* site via homologous recombination. Secondly, two strains SIPI-3927-C1 and C2 were also constructed by integrating halogenase genes *vcm8* and *orf10* into artificial *attB* sites of SIPI-3927-*attB*, respectively. Meantime, three strains SIPI-3927-C3, C4 and C5 containing overexpressing glycosyltransferase A, B and C genes were obtained, respectively. Through fermentation analyses, the results showed that SIPI-3927-C1 and C2 could increase A82846B ratios, in which SIPI-3927-C1 showed a better performance. Moreover, the titer of SIPI-3927-C3 was highest in those of three strains. Finally, the strain SIPI-3927-C6 was constructed by integrating both *orf10*-encoded halogenase and *orf11*-encoded glycosyltransferase A, of which the yield and ratio of A82846B in shake-flask fermentation reached 1200 mg/L and 73.6%, respectively. Besides, the yield and ratio of A82846B in SIPI-3927-C6 grew up to 2520 mg/L and 86.5% in the 5-L fermenter culture, respectively. In conclusion, overexpressing *orf10* gene can increase A82846B ratio,while overexpressing *orf11* gene can increase A82846B titer as well. The artificial *attB* site is effective for inserting new genes.

## Key points

- It is first time to construct engineering strains of *K. aridum* for A82846B production.
- The *attB*-site has inserted into *K. aridum* SIPI-3927 by homologous recombination.
- The double-gene overexpressing engineered strain was constructed.
- The highest titer of A82846B was obtained in *K. aridum* SIPI-3927-C6.

## Introduction

Oritavancin is a semi-synthetic antibiotic, which was recently approved by the Food and Drug Administration (FDA) (Brade et al. 2016; Corey et al. 2014) for treatment of gram-positive pathogens induced Acute Bacterial Skin and Skin Structure Infections (ABSSSIs) and Methicillin-resistant *Staphylococcus Aureus* (MRSA) (Edelsberg et al. 2014; Kaatz et al. 1998; Rupp et al. 2001). Compared with previous glycopeptide antibiotics, Oritavancin has a prolonged half-life about 245 h, which allows a 7-day course of treatment for one single dose (Brade et al. 2016).

Oritavancin is chemically synthesized by adding a 4-(4-chlorophenyl) benzyl group to A82846B via reductive alkylation (Leadbetter et al. 2010). A82846B, along with two structurally similar components A82846A and A82846C, is produced by *Nocardia*, *Amycolatopsis*, *Kibdelosporangium* (Rafai et al. 2016). Since A82846A, A82846B, and A82846C only differ in their chlorine atom number, the purification of these compounds requires very cumbersome steps (Hamill et al. 1998), which definitely decreases the recovery ratio. Furthermore, the probable side effects of those impurities make it more necessary to reduce ratios of A82846A and C in fermentation broth.

The biosynthetic pathway of A82846B, including a total of 39 putative genes, was firstly reported in *A. orientalis* NRRL 18098 (Van Wageningen et al. 1998). The current dilemma was that halogenase encoded *orf10* gene activity was not high enough (NCBI Accession No: AJ223998.1). It was proposed that the catalytic activity of halogenase was not sufficient for complete halogenation of A82846. Therefore, improving the activity of halogenase may be a good strategy to solve this predicament. Wang et al. (2018) tried to overexpress the three-copy halogenase coding gene in *A. orientalis* SIPI-18099, and successfully increased the A82846B yield as well as the purity in shake-flask. This result partially demonstrated the hypothesis of insufficient halogenase activity accounting for incomplete halogenation of A82846A and A82846C and enhancement of halogenase activity can help to solve this problem.

**Fig. 1:**
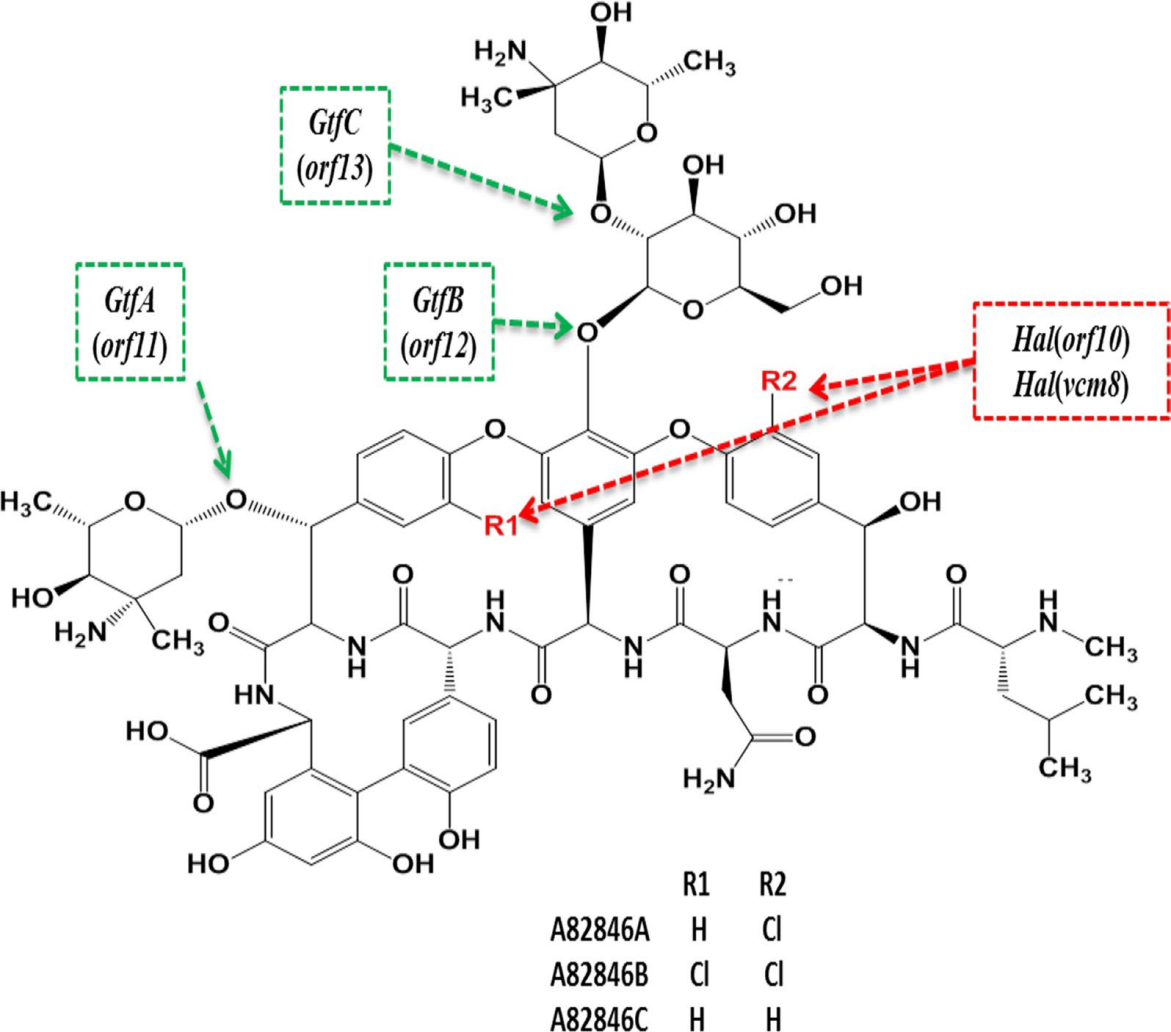
Structure and the key biosynthesis gene of A82846. Green box is a glycosyltransferase reaction site; red box is a halogenase reaction site; encoded gene of enzyme in brackets

*Kibdelosporangium aridum*, a natural A82846B producer, has a higher yield (900 mg/L) and ratio (34%) of A82846B in shake-flask compared with *A. orientalis*. Thus, overexpression of halogenase gene was more promising to generate a better performed high-yield A82846B producer of *K. aridum*. The genes coding glycosyltransferases involved in the biosynthesis of various glycopeptide antibiotics were predicted to be used to link heptapeptide and three glycosyl groups in A82846B biosynthesis (Van Wageningen et al. 1998). It was found that glycosyltransferases (*GtfA*, *GtfB*, *GtfC*, reaction site as Fig. 1) were encoded by *orf11*, *orf12* and *orf13* (NCBI Accession No: AJ223998.1), and could have a positive regulatory effect on the biosynthesis of A82846B (Solenberg et al. 1997).

The *attB/P* integration system was widely used in *Streptomyces*, which depends on homologous recombination between the *attB* site and the *attP* site mediated by the ΦC31 integrase (Patricia et al. 2002). This system allows exogenous genes to be integrated into chromosome and expressed stably and efficiently (Yuan et al. 2016; Lee et al. 2016). In this study, we provide a shortcut for recombinant strains construction by inserting an artificial *attB* site into *K. aridum* chromosome via homologous recombination. Based on the engineered chassis, the co-expression of *orf10* coding halogenase and *orf11* coding glycotransferase led to significant boost of A82846B titer and ratio. The constructed engineering strains can be reducing the industrialization cost of A82846B. This strategy of constructing engineered strains can provide a new method for other strains that lack similar attB sites.

## Materials and methods

### Strains, plasmids, and primers

Bacterial strains and plasmids used in this study are listed in Table 1; primers are listed in Table 2.

**Table 1:** Bacterial strains and plasmids

Strains or plasmids	Description	Source
Plasmid vectors		
pSET152	*acc(3)IV*, *oriTRK2*, *φC31*, *int*	Bierman et al. (1992)
pKC1139	*acc(3)IV*, *pSG5*, *oriTRK2*	Bierman et al. (1992)
pSBJ153	*acc(3)IV*, *oriTRK2*, kasO*P promoter	Wang et al. (2013)
pKC1139-*attB*	*acc(3)IV*, *pSG5*, *oriTRK2*, *attB*	This study
pSET152-*vcm8*	*acc(3)IV*, *oriTRK2*, Apra^r^, kasO*P, *vcm8*	This study
pSET152-*orf10*	*acc(3)IV*, *oriTRK2*, Apra^r^, kasO*P, *orf10*	This study
pSET152-*orf11*	*acc(3)IV*, *oriTRK2*, Apra^r^, kasO*P, *orf11*	This study
pSET152-*orf12*	*acc(3)IV*, *oriTRK2*, Apra^r^, kasO*P, *orf12*	This study
pSET152-*orf13*	*acc(3)IV*, *oriTRK2*, Apra^r^, kasO*P, *orf13*	This study
pSET152-*orf10*–*orf11*	*acc(3)IV*, *oriTRK2*, Apra^r^, kasO*P, *orf10*–*orf11*	This study
Strains		
*E. coli* DH5α	Host for general cloning	FENGHBIO, China
*E. coli* ET12567/pUZ8002	Donor strain for intergenetic conjugation between *E. coli* and Streptomyces, Kan^r^, Chl^r^	FENGHBIO, China
*A. orientalis* ATCC 43491	Vancomycin-producing the parental strain	ATCC
*K. aridum* SIPI-3927 (CGMCC 4.7675)	A82846B-producing strain	CGMCC
SIPI-3927-*attB*	SIPI-3927, *attB*	This study
SIPI-pSET152	SIPI-3927-*attB*, pSET152	This study
SIPI-3927-C1	SIPI-3927-*attB*, pSET152-*vcm8*	This study
SIPI-3927-C2	SIPI-3927-*attB*, pSET152-*orf10*	This study
SIPI-3927-C3	SIPI-3927-*attB*, pSET152-*orf11*	This study
SIPI-3927-C4	SIPI-3927-*attB*, pSET152-*orf12*	This study
SIPI-3927-C5	SIPI-3927-*attB*, pSET152-*orf13*	This study
SIPI-3927-C6	SIPI-3927-*attB*, pSET152-*orf10*–*orf11*	This study

**Table 2:** Primers used in this study

Primers	Purpose	Sequence (5′–3′)
*orf35*-*attB*-F*/*R	Amplification of *orf35*-*attB* for construction of pSET1139-*attB*	CTGCAGGTCGACTCTAGACCTCGATCCGGACCACGAGCAC *CCCGGGGAGCCCAAGGGCACGCCCTGGCACCCGCACCGCGG*TCAATATCCGTACCGGCT
*orf36*-*attB*-F*/*R	Amplification of *orf36*-*attB* for construction of pSET1139-*attB*	*TGCCAGGGCGTGCCCTTGGGCTCCCCGGGCGCGTACTCCAC*TCACCGGTCGATCAAGGG GCGCGCGGCCGCGGATCCCGAGACGCTGACCACTGGGCAG
kasO*P-F/R	Amplification of kasO*P promoter for construction of pSET152-*vcm8*	CAAGCTTGGGCTGCAGGTCGACTCTAGATGTTCACATTCGAACCGTCTC ACCACATCGAAGTCTTCGACCGACATATGGACACTCCTTACTTAGACTG
*vcm8*-F*/*R	Amplification of *vcm8* for construction of pSET152-*vcm8*	GTCTAAGTAAGGAGTGTCCATATGTCGGTCGAAGATTTCGATGTTGTG TCGATATCGCGCGCGGCCGCGGATCCTCAGGCCGGGTGGTGCGGCAGC
*orf10*-F*/*R	Amplification of *orf10* for construction of pSET152-*orf10*	CAGTCTAAGTAAGGAGTGTCCATATGTCGGTCGAAGACTTCGATGT TCGATATCGCGCGCGGCCGCGGATCCTCATGCCGGATGGTGCGGCAGCC
*orf11*-F*/*R	Amplification of *orf11* for construction of pSET152-*orf11*	GTCTAAGTAAGGAGTGTCCATATGCGCGTGTTGATTACGGGGTGTG TCGATATCGCGCGCGGCCGCGGATCCTCAGGCGGGAACAGTCGGC
*orf12*-F*/*R	Amplification of *orf12* for construction of pSET152-*orf12*	GTCTAAGTAAGGAGTGTCCATATGCGTGTGCTGTTGGCGACGTGTG TCGATATCGCGCGCGGCCGCGGATCCTTACGCGGAAACAGTCGGC
*orf13*-F*/*R	Amplification of *orf13* for construction of pSET152-*orf13*	GTCTAAGTAAGGAGTGTCCATATGCGTGTGTTGTTGTCGACGGCTG TCGATATCGCGCGCGGCCGCGGATCCTTACGCGAGAACAGCCGAC
*orf10*–*orf11*-F*/*R	Amplification of *orf10*–*orf11* for construction of pSET152-*orf10*–*orf11*	GTCTAAGTAAGGAGTGTCCATATGTCGGTCGAAGACTTCGATGT TCGATATCGCGCGCGGCCGCGGATCCATGTCAGGCGGGAACAGTCGGCTTTT
*attB*-*F/R*	Amplification of artificial *attB site* in *K. aridum*	CCGCGGTGCGGGTGCCAGGGCGTGC CTCTCCCCTCTGATCGAGGACCAG

The Italicized part represents *attB* sequence; underline represents restriction enzyme cutting site

### DNA manipulation

The manipulations of genomic DNA, plasmid DNA isolation, restriction endonuclease digestion, and DNA ligation were performed according to standard procedures (Sambrook and Russell 2001). The enzymes were purchased from Takara, Japan and Thermo Fisher Scientific (Thermo, USA). ClonExpress MultiS One Step Cloning Kit (CMOSTK) was purchased from Vazyme Biotech Co., Ltd. The A82846B standard was purchased from MedKoo Biosciences, Inc. The defoamer of SAG471 was purchased from Beijing BaiYuan Chemical Co., Ltd. All chemicals used were molecular biology grade and commercially available.

### Construction of plasmids

In order to construct pKC1139-*attB* plasmid containing *attB* site, *orf35*-*attB*-F/R and *orf36*-*attB*-F/R were used as primers, respectively, and SIPI-3927 genome was used as a template to obtain two 1000 bp homology arms by PCR amplification. This PCR products and pKC1139 plasmid with BamH1/Xba1 restriction enzyme digestion were ligated by CMOSTK to obtain a pKC1139-*attB* plasmid.

The pSET152 linear vector was obtained by digesting the pSET152 plasmid with Xba1/BamH1 restriction enzyme. The kasO*P promoter was amplified by PCR using kasO*P-F/R as primers and plasmid pSBJ153 as a template. The *vcm8* (NCBI Accession No: HQ679900.1) was amplified by PCR using *vcm8*-F/R as primers and strain ATCC43491 as template. These three products were then ligated by CMOSTK to obtain a pSET152-*vcm8* plasmid.

The *orf10*, *orf11*, *orf12*, *orf13* and *orf10*–*orf11* productions were amplified, which SIPI-3927 genome as a template, and *orf10*-F/R, *orf11*-F/R, *orf12*-F/R, *orf13*-F/R and *orf10*–*orf11*-F/R as primers, respectively. The pSET152-*vcm8* plasmid with Nde1/BamH1 restriction enzyme digestion was used as a linear vector. The linear vector and the PCR products which we amplified above were ligated by CMOSTK to obtain plasmids pSET152-*orf10*, pSET152-*orf11*, pSET152-*orf12*, pSET152-*orf13* and pSET152-*orf10*–*orf11*, respectively.

### Construction of recombinant strains K. aridum

The plasmids were introduced into *K. aridum* by the *E. coli*–*Streptomyces* conjugation method described previously (Kieser et al. 2000). The *E. coli* ET12567/pUZ8002 containing the plasmid was grown in the presence of antibiotics (50 mg/L apramycin, 25 mg/L chloramphenicol, and 50 mg/L kanamycin) to an OD600 of 0.4–0.6. The cells were washed twice with an equal volume of LB medium, and resuspended in 2 mL LB medium. The *K. aridum* was incubated in 30 mL YEME medium (yeast extract 0.3%, tryptone 0.5%, malt extract 0.3%, sucrose 34%) at 30 °C for 48 h, then 10% of the culture was transferred into 30 mL YEME medium for 36 h growth. Mycelium was collected by centrifugation and resuspended in 2 mL LB. 2 mL ET12567/pUZ8002 cells mixed with the resuspended *K. aridum*, and spread the mixture on MISP-4 plates (mannitol 0.5%, glucose 0.1%, soluble starch 0.5%, soybean cake powder 0.5%, tryptone 0.2%, yeast extract 0.1%, ammonium sulfate 0.2%, sodium chloride 0.1%, dipotassium hydrogen phosphate 0.1%, carbonic acid calcium 0.2%, trace element 1 mL, agar powder 2% and pH 6.8–7.0). The plates were incubated for 16–20 h at 30 °C and then overlaid with 1 mL water containing 400 μg nalidixic acid and 800 μg apramycin. The plates were incubated again for 7–12 days at 30 °C until the recombinant strains were obtained. The recombinants were then transferred onto slant medium containing 50 mg/L apramycin.

Firstly, the pKC1139-*attB* plasmid was transferred into *K. aridum* SIPI-3927 as described above. Secondly, the *K. aridum* SIPI-3927-*attB* recombinant strain with loss of apramycin resistance was obtained by double exchange screening. Finally, the other pSET152 plasmids were transferred to *K. aridum* SIPI-3927-*attB* as described above to obtain different recombinant strains.

### Culture of *K. aridum*

The parental type or recombinant *K. aridum* strains were grown on solid medium (glucose 1%, starch 2%, yeast extract 0.5%, hydrolyzed casein 0.5%, calcium carbonate 0.1%, agar 2%, pH 6.8–7.0) at 30 °C for 6–7 days. For fermentation culture (Tian et al. 2020), a 1 cm * 2 cm agar piece was transferred into a 250-mL flask with 30 mL seed medium (glucose 2%, starch 0.5%, corn syrup 0.5%, yeast powder 0.5%, ammonium sulfate 0.5%, and calcium carbonate 0.5%, pH 6.8) and incubated at 30 °C, 220 rpm for 48 h. Then 10% seed culture was inoculated into a 250-mL flask with 30 mL production medium (glucose 6%, corn starch 1%, hydrolyzed casein 0.5%, soy flour 1%, yeast powder 0.5%, beef extract 1%, potassium dihydrogen phosphate 0.05%, magnesium sulfate heptahydrate 0.018%, sodium chloride 0.3%, calcium carbonate 0.5%, SAG471 defoamer 0.03%, pH 6.6–6.8) and incubated at 34 °C, 250 rpm for 144 h. Shake-flask fermentations were carried out in three independent experiments.

### Production of A82846B in 5-L fermenter

To research A82846 production, 2 cm * 3 cm agar pieces of the *K. aridum* SIPI-3927 or SIPI-3927-C6 from slant medium were cultured in 750-mL flasks with 100 mL seed medium at 30 °C, 220 rpm, for 48 h. Then 10% seed culture was inoculated into 3 L of production medium in a 5-L fermenter. The fermentation temperature was kept at 34 °C, and the pH was adjusted to 6.8 with NaOH at the beginning. 30% glucose solution was added when the content of glucose dropped below 1.0%, and its concentration was kept at about 1–2% during the fermentation period.

### Analytic method

One milliliter of the culture was centrifuged at 12,000 rpm for 20 min to remove the precipitate, and then the supernatant was diluted 5- to 20-fold. Processed sample was assayed by HPLC with a gradient elution program (0 → 20 min, A:B = 95:5 → 80:20; 20 → 22 min, A:B = 80:20 → 95:5; 22 → 27 min, A:B = 95:5, A: 0.1% trifluoroacetic acid, B: acetonitrile) in a phenyl chromatographic column (4.6 × 250, 5 μm, Welch, China) with at 1.0 mL/min and detection at 225 nm.

To measure biomass, 10 mL fermentation culture was centrifuged in a graduated centrifuge tube for 10 min, 5000 rpm. Measure the volume of supernatant (v), the value of (10 − v)/10 was the biomass which could reflect the growth conditions of the strains.

### Data analysis

The data were analyzed using Excel 2010, SPSS 20.0, OriginPro8.5 data analysis and statistical software.

## Results

### Construction of *K. aridum* SIPI-3927-attB strain containing attB site

**Fig. 2:**
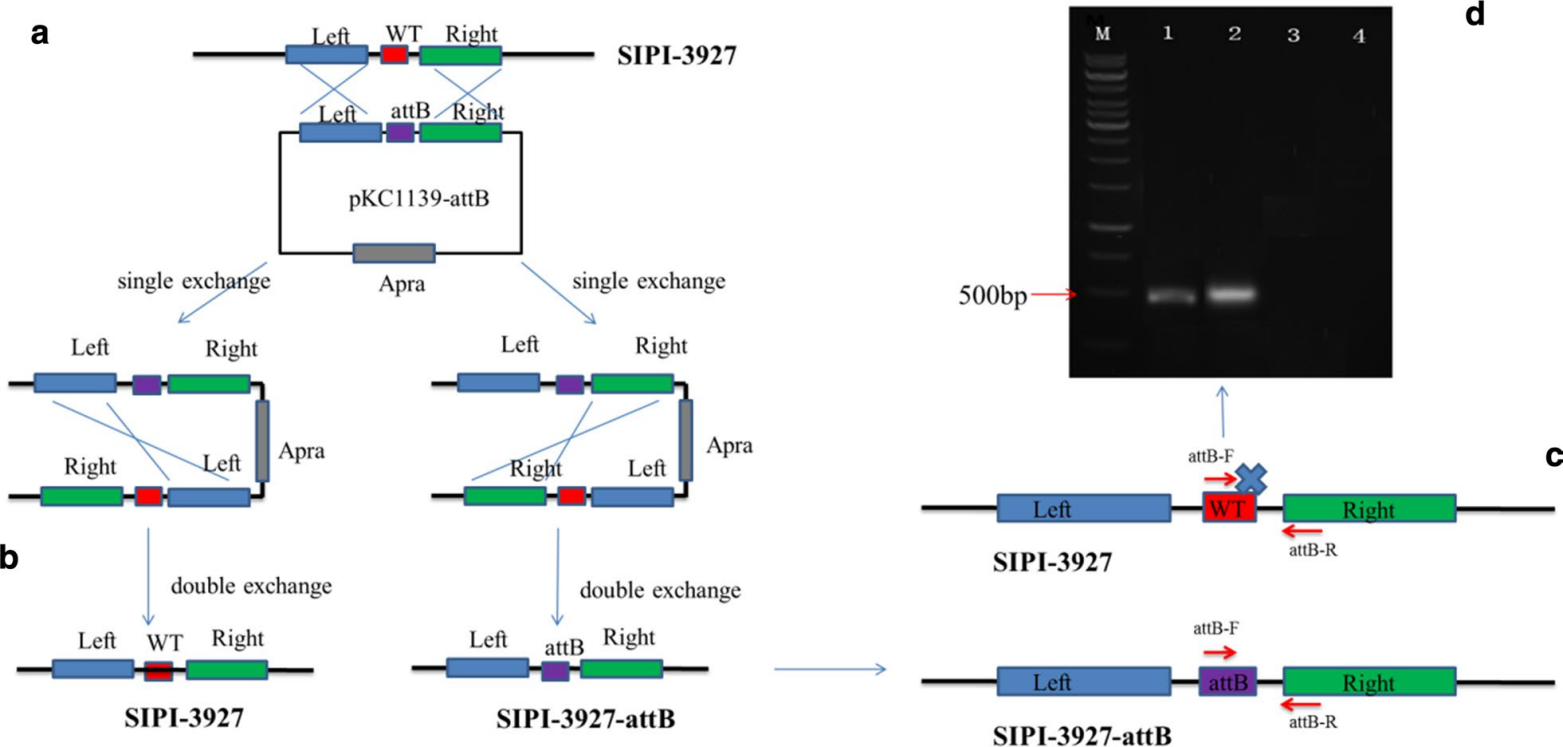
Homologous recombination to construct SIPI-3927-*attB* strain containing *attB* site. **a** Single exchange screening of recombinant strains containing apramycin resistance. **b** Double exchange screening of recombinant strains with loss of apramycin resistance. **c**, **d** PCR amplification results of double exchange strains. M, maker; 1–2, double-exchange strain containing *attB* site; 3, double-exchange strain without *attB* site; 4, parent strain

**Fig. 3:**
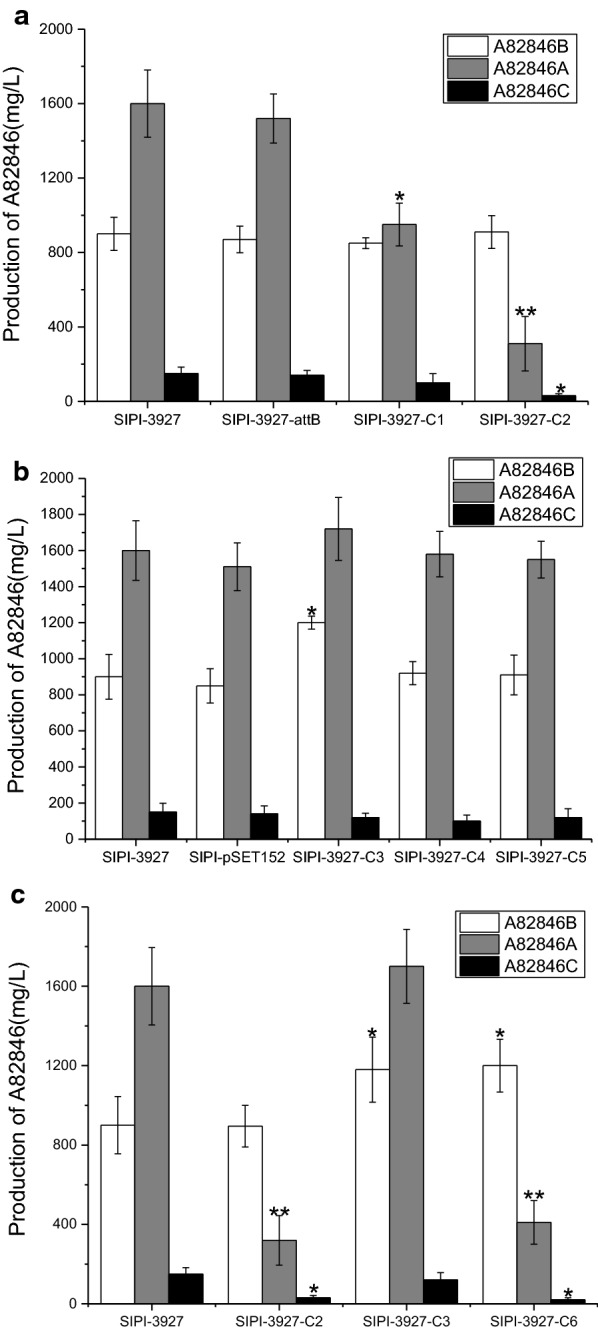
**a** Effects of different strains containing halogenase on yield of A82846 analogs. **b** Effects of different strains containing glycosyltransferase on yield of A82846 analogs. **c** Comparison of yield of A82846 analogs in control strain, SIPI-3927-C2, SIPI-3927-C3 and SIPI-3927-C6. Different asterisks represent significant differences compared to the parent strain SIPI-3927 (*P < 0.05; **P < 0.01)

Conjugal transfer is the main method of molecular manipulation in *Streptomyces*. Because there is no *attB* site in *K. aridum*, it is difficult to insert an exogenous gene into the genome through the ΦC31 integrase (Kim et al. 2008). To solve this predicament, we constructed an *attB* site into the chromosome of *K. aridum* by homologous recombination (Fig. 2a, b). The single-exchange transformants were generated at several times, and a total of 580 transformants were selected for obtaining the second homologous recombination. Three strains were obtained without apramycin resistance by double-exchange. The double-exchange efficiency was about 0.5%. The product was verified thought PCR amplification procedure which three strains were used as a template and the *attB*-R/F as primers (Fig. 2c, d). Finally, we obtained two strains containing the *attB* site, and 1# was named as *K. aridum* SIPI-3927-*attB*. In addition, the *attB* site insertion has no significant effect on the biosynthesis of A82846B (Fig. 3a).

### Overexpression of orf10 and vcm8 in K. aridum SIPI-3927-attB

In the biosynthesis of A82846B, due to insufficient expression of the halogenase, the impurities A82846A and A82846C have more ratio than A82846B in whole A82846. In order to increase the ratio of A82846B, we overexpressed the halogenase gene. Two plasmids pSET-*vcm8* and pSET-*orf10* were constructed which controlled by a strong promoter kasO*P. They contained the extra copy gene of *vcm8* and *orf10*, respectively. Subsequently, these plasmids were inserted into the *K. aridum* SIPI-3927-*attB* by conjugate transformation. Then, the recombinant strains of SIPI-3927-C1 and SIPI-3927-C2 were obtained by resistance selection with 50 μg/mL apramycin. The result showed that there was no significant difference about A82846B yields among strains of SIPI-3927-*attB*, SIPI-3927-C1, SIPI-3927-C2 and the parental strain (Fig. 3a), of which the ratio of SIPI-3927-C2 for A82846A, B, and C were 24.8%, 72.8%, and 2.4%, respectively, which were significantly different from the parental strain SIPI-3927 (60.4%, 34%, and 5.6%). Meanwhile, the ratio of A82846B in the SIPI-3927-C1 was increased to 44.7%. These results suggest that the insertion of *K. aridum orf10* gene is more efficient than *A. orientalis vcm8* gene during enhancing the ratio of A82846B.

### Effect of orf11, orf12 and orf13 genes encoded glycosyltransferases on A82846B production

Glycosyltransferases were catalytic enzymes that linked the heptapeptide and three glycosyl groups in the A82846B biosynthesis. The *GtfA*, *GtfB* and *GtfC* of A82846B biosynthesis were encoded by the *orf11*, *orf12* and *orf13*, respectively. Three plasmids, pSET152-*orf11*, pSET152-*orf12* and pSET152-*orf13*, containing the extra gene of *orf11*, *orf12* and *orf13*, respectively, controlled by a strong promoter kasO*P were constructed. These plasmids were transferred into the SIPI-3927-*attB* strain by conjugative transfer. Finally, we obtained recombinant strains of SIPI-3927-C3, SIPI-3927-C4 and SIPI-3927-C5. As shown in Fig. 3b, these recombinant strains of A82846B titer were 1100 mg/L, 920 mg/L and 910 mg/L, respectively. In SIPI-3927-C3, the yield of A82846B was increased by 1.22-fold compared with parental type strain SIPI-3927. In addition, it was found that the *Gtfs* did not increase the ratio of A82846B. The results showed that the *orf11* gene can significantly increase the yield of A82846B.

### Effect of orf10–orf11 on A82846B yield and ratio

**Fig. 4:**
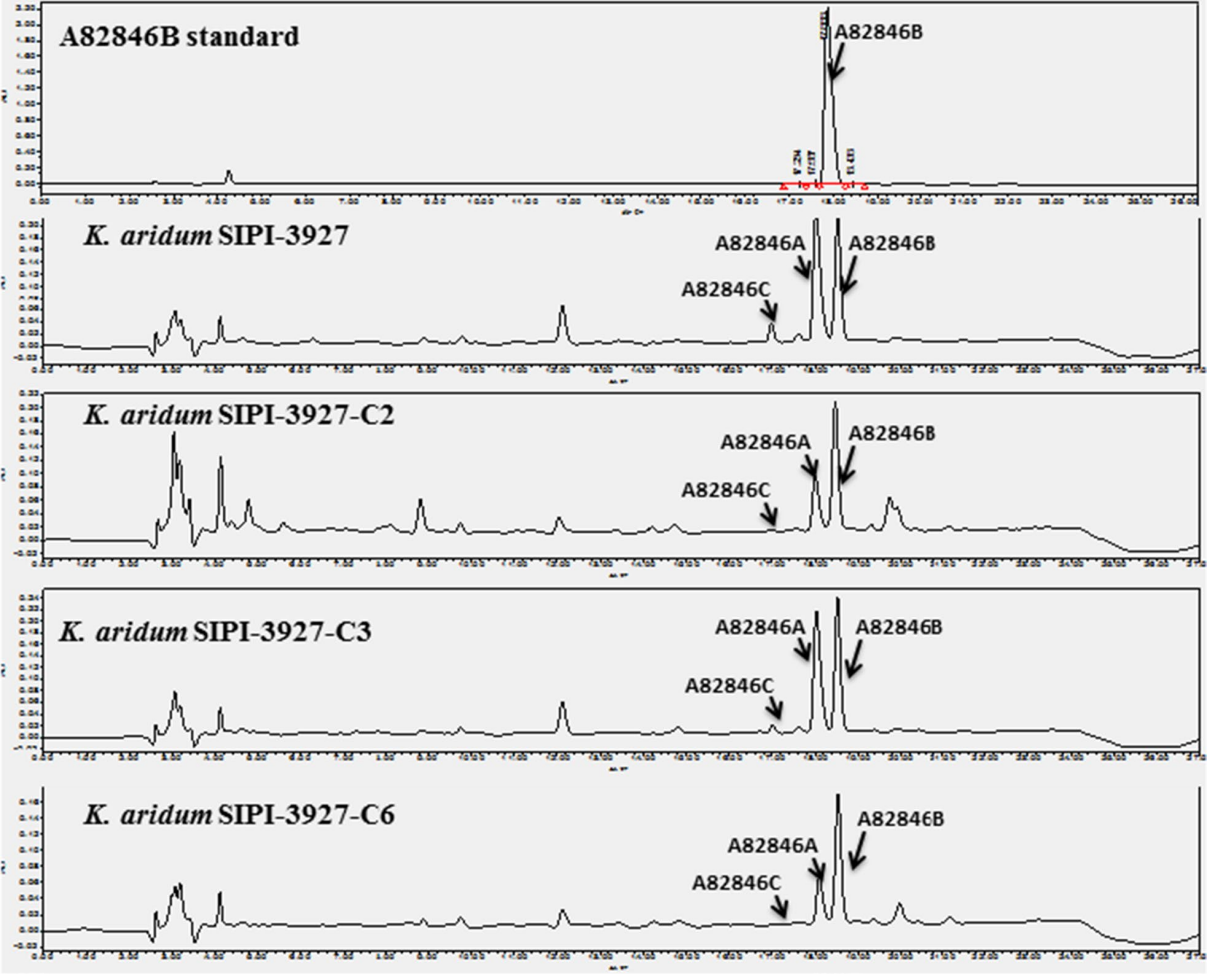
HPLC analysis of the A82846 components production in fermentation broths

All above studies indicated that *orf10* and *orf11* can increase the ratio and yield of A82846B, respectively. These were positive regulatory genes for A82846B biosynthesis. The pSET152-*orf10*–*orf11* plasmid was constructed to increasing yield and ratio of A82846B, which controlled by a strong promoter kasO*P. The plasmid was transferred into the SIPI-3927-*attB* strain by conjugative transfer, then the recombinant strain SIPI-3927-C6 was obtained. It can be seen that *orf10* significantly improves the A82846B ratio, while *orf11* significantly increases the A82846B yield in the fermentation shake-flask (Fig. 4). Compared with the parental strain SIPI-3927, the A82846B yield in strain SIPI-3927-C6 was increased from the initial 930 mg/L to 1200 mg/L, and the A82846B ratio was increased from the initial 34.0% to 73.6% (Fig. 3c). The impurities A82846A and A82846C decreased from 60.4% and 5.6% to 25.2% and 1.2%, respectively (Fig. 3c). The results indicated that overexpression of the *orf10*–*orf11* in the parental strain is an efficient method for reducing impurities and increasing yield.

### Shake-flask fermentation stability of recombinant strain SIPI-3927-C6

Since the recombinant strains overexpresses itself genes of *orf10* and *orf11*, the extra gene loss could be caused by multiple generation culture. It is necessary to study the stability of the recombinant strain SIPI-3927-C6 in shake-flask fermentation. SIPI-3927-C6 was generated to the F6 in solid medium, and the A82846B production of the F1–F6 strains were fermented by shake-flask. The A82846B yields of the F1–F6 strains were all above 1000 mg/L, and the highest titer was 1253 mg/L (Table 3). The ratios of A82846B were above 70.3%, and the highest ratio of A82846B was 74.1% (Table 3). The results indicated that SIPI-3927-C6 has good genetic stability.

**Table 3:** Comparison recombinant F1–F6 strains of SIPI-3927-C6 in shake-flask fermentation

Generation	Biomass (%)	Yield of A82846B (mg/L)	A82846B (%)
F1	22	1025	70.3
F2	24	1201	73.6
F3	25	1253	72.4
F4	23	1198	74.1
F5	24	1122	73.9
F6	22	1234	73.5

### Culture of *K. aridum* SIPI-3927-C6 and SIPI-3927 in 5-L fermenter

**Fig. 5:**
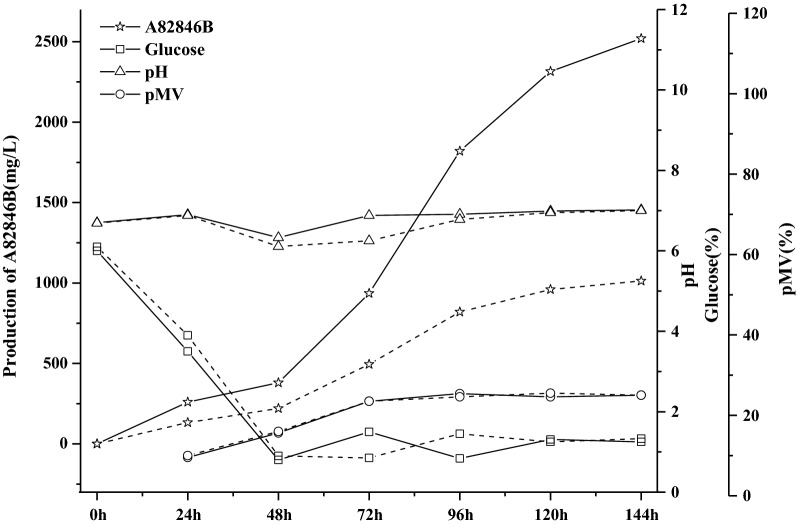
Comparison metabolic curves between recombinant strain SIPI-3927-C6 and parent strain SIPI-3927 in 5L fermentor. Solid line, SIPI-3927-C6; dotted line, SIPI-3927

The A82846B production by *K. aridum* SIPI-3927-C6 and SIPI-3927 were studied in a 5-L fermenter. During the culture process, the glucose was rapidly consumed within 48 h in *K. aridum* strains, and then the glucose concentration was controlled between 1–2% by 30% glucose streaming (Fig. 5). At about 144 h, the A82846B yields of SIPI-3927 and SIPI-3927-C6 reached 1013 mg/L (A82846B ratio was 38.9%) and 2520 mg/L (A82846B ratio was 86.5%), respectively (Table 4). The A82846B yield of the recombinant strain SIPI-3927-C6 was 2.5-fold higher than the parental strain SIPI-3927. The ratio of A82846A and A82846C decreased to 13.4% and 0.1%, respectively, compared with that of SIPI-3927 (A82846A was 52.1% and A82846C was 9.0%). This change of A82846 ratio drastically reduced the purification pressure, providing a low-price substrate for the alkylation synthesis of Oritavancin. Therefore, the construction of the recombinant *K. aridum* SIPI-3927-C6 was an important method to reduce the cost of A82846B production.

**Table 4:** Comparison the yield and ratio of A82846 in 5-L fermentation

Strains	A82846B (%)	A82846A (%)	A82846C (%)	Yield of A82846B (mg/L)
SIPI-3927	38.9	52.1	9.0	1013
SIPI-3927-C6	86.5	13.4	0.1	2520

## Discussion

Conjugal transfer was an important method in *E. coli*–*Streptomyces* system, which can rapidly inserted genes into *Streptomyces* by gene overexpression (Bierman et al. 1992). Since exogenous genes had genetic instability in *Streptomyces*, it could be solved by the *attB/P* integration system. The *attB/P* integration system inserted exogenous genes into the genome of *Streptomyces*, which enables more stable heredity in multiple generations (Olano et al. 2008). However, the absence of natural *attB* site in *K. aridum* chromosome impeded the exogenous genes overexpression and genetic engineering of *K. aridum*, it was not possible to insert the integrin plasmid of pSET152 into *K. aridum*. In our study, we constructed an *attB* site on the genome of *K. aridum* by homologous recombination. The strain allows the pSET152 plasmid to quickly integrate the exogenous gene into the chromosome. Compared with Wang’s (2018) research, this genetic manipulation strategy could insert more *attB* sites into the genome for further study, which may greatly improve the overexpression ability in *K. aridum*, and the passage of the strain was more stable. It was the first time to propose a new strategy to recombinant DNA of the A82846B in the *K. aridum*.

Van Wageningen et al. (1998) and Xu et al. (2014) reported that halogenases of A82846B and vancomycin were encoded by *orf10* and *vcm8*, respectively. Meantime, the *vcm8* encoded halogenase may be theoretically eliminated the A82846A and A82846C impurities in current industrial vancomycin production, given the fact that vancomycin has two chlorine substitutes without any mono- or non-chlorine analogs. However, the experimental results showed that the halogenase encoded by the orf10 gene may specifically increase the halogenation ability in the biosynthesis of A82846B. Therefore halogenase of vancomycin was not an optimal choice on the biosynthesis of A82846B. Meanwhile, Solenberg et al. (1997) proposed that glycosyltransferases have a positive effect on increasing the production of A82846B. In our study, it is confirmed that *orf11*-encoded *GtfA* can effectively increase the yield of A82846B.

The yield and ratio of A82846B in 5-L fermenter reached 2520 mg/L and 86.5% by supplying the sufficient oxygen and carbon source compared with shake-flask fermentation in SIPI-3927-C6. It was evidenced that oxygen and glucose control may be a key factor in the production of A82846B. Similarly, Wang et al. (2018) constructed a recombinant strain, *A. orientalis chal*-3, containing three consecutive copies of halogenase encoding halogenase of A82846B biosynthesis, the yield and ratio of A82846B were reached 2200 mg/L and 88.2% in 5 L fermentor culture, respectively. Compared with Wang’s study, our research improved by 14.5% A82846B yield with almost the same A82846B ratio. Moreover, the fermentation yield and ratio were remained stable during culture for six consecutive generations. According to industrial production of recombinant strains, it was documented that continuous multiple-copy gene easily lost during generation due to secondary recombination (Myronovskyi et al. 2018; Olano et al. 2008). This method could insert more *attB* sites in the genome of *K. aridum*, which greatly avoids gene loss and improves applicant potential of our strain in commercial A82846B production. In addition, the fermentation process optimization of the engineering strain SIPI-3927-C6 was another key factor for the A82846B production in the future. The regulation of the fermentation process will become the focus of our next research.

In conclusion, it was the first time to constructing an artificial *attB* site in *K. aridum* and overexpression of *orf10*–*orf11*, and more *attB* sites can be inserted into the genome for further research. Furthermore, the recombinant strain SIPI-3927-C6 could be used for the industrial production of A82846B, and it was shown a great significance in reducing the production cost of Oritavancin.

## Data Availability

Not applicable.

## References

[CR1] Bierman M, Logan R, O’Brien K, Seno ET, Rao RN, Schoner BE (1992). Plasmid cloning vectors for the conjugal transfer of DNA from *Escherichia coli* to *Streptomyces* spp.. Gene.

[CR2] Brade KD, Rybak JM, Rybak MJ (2016). Oritavancin: a new lipoglycopeptide antibiotic in the treatment of gram-positive infections. Infect Dis Ther.

[CR3] Corey GR, Kabler H, Mehra P, Gupta S, Overcash JS, Porwal A, Giordano P, Lucasti C, Perez A, Good S, Jiang H, Moeck G, O’Riordan W (2014). Single-dose oritavancin in the treatment of acute bacterial skin infections. N Engl J Med.

[CR4] Edelsberg J, Weycker D, Barron R, Li X, Wu H, Oster G, Badre S, Langeberg WJ, Weber DJ (2014). Prevalence of antibiotic resistance in US hospitals. Diagn Microbiol Infect Dis.

[CR5] Hamill RL, Mabe JA, Mahoney DF, Nakatsukasa WM, Yao RC (1998) A82846 antibiotics. US Pat No. 005843437A

[CR6] Kaatz GW, Seo SM, Aeschlimann JR, Houlihan HH, Mercier RC, Rybak MJ (1998). Efficacy of LY333328 against experimental methicillin-resistant *staphylococcus aureus* endocarditis. Antimicrob Agent Chemother.

[CR7] Kieser T, Bibb MJ, Buttner MJ, Chater K, Hopwood DA (2000). Practical streptomyces genetics: a laboratory manual.

[CR8] Kim MK, Ha HS, Choi SU (2008). Conjugal transfer using the bacteriophage ϕC31 *att/int* system and properties of the *attB* site in *Streptomyces ambofaciens*. Biotechnol Lett.

[CR9] Leadbetter MR, Linsell MS, Lee J, Liu J (2010) Process for preparing glycopeptide phosphonate derivatives. US Pat No. 7728104B2

[CR10] Lee KS, Lee BM, Ryu JH, Kim DH, Kim YH, Lim SK (2016). Increased vancomycin production by overexpression of *MbtH*-like protein in *Amycolatopsis orientalis* KFCC10990P. Lett Appl Microbiol.

[CR11] Myronovskyi M, Rosenkränzer B, Nadmid S, Pujic P, Normand P, Luzhetskyy A (2018). Generation of a cluster-free *Streptomyces albus* chassis strains for improved heterologous expression of secondary metabolite clusters. Metab Eng.

[CR12] Olano C, Lombó F, Méndez C, Salas JA (2008). Improving production of bioactive secondary metabolites in *Actinomycetes* by metabolic engineering. Metab Eng.

[CR13] Patricia C, Rob T, Sally B, Smith MCM (2002). The streptomyces genome contains multiple pseudo-attB sites for the ΦC31-encoded site-specific recombination system. J Bacteriol.

[CR14] Rafai FA, Krishna G, Ding M, Chemburkar SR, Knable CM, Petzel JJ, Pruyne JJ, Reamer DM (2016) High purity Oritavancin and method of producing same. US Pat No. 9649352B2

[CR15] Rupp ME, Fey PD, Longo GM (2001). Effect of LY333328 against vancomycin-resistant enterococcus faecium in a rat central venous catheter-associated infection model. J Antimicrob Chemother.

[CR16] Sambrook J, Russell DW (2001). Molecular cloning: a laboratory manual.

[CR17] Solenberg PJ, Matsushima P, Stack DR, Wilkie SC, Thompson RC, Baltz RH (1997). Production of hybrid glycopeptide antibiotics in vitro and in *Streptomyces toyocaensis*. Chem Biol.

[CR18] Tian X, Gao P, Liu Y, Hu H (2020). Breeding of the A82846B-producing strains and optimization of its fermentation media. Chin J Pharm.

[CR19] Van Wageningen AM, Kirkpatrick PN, Williams DH, Harris BR, Kershaw JK, Lennard NJ, Jones M, Jones SJ, Solenberg PJ (1998). Sequencing and analysis of genes involved in the biosynthesis of a vancomycin group antibiotic. Chem Biol.

[CR20] Wang W, Li X, Wang J, Xiang S, Feng X, Yang K (2013). An engineered strong promoter for *streptomycetes*. Appl Environ Microb.

[CR21] Wang W, Yang S, Wu Y, Shen X, Chen S (2018). Enhancement of A82846B yield and proportion by overexpressing the halogenase gene in *Amycolatopsis orientalis* SIPI18099. Appl Microbiol Biotechnol.

[CR22] Xu L, Huang H, Wei W, Zhong Y, Tang B, Yuan H, Zhu L, Huang W, Ge M, Yang S, Zheng H, Jiang W, Chen D, Zhao G, Zhao W (2014). Complete genome sequence and comparative genomic analyses of the vancomycin-producing *Amycolatopsis orientalis*. BMC Genom.

[CR23] Yuan P, Zhou R, Chen X, Luo S, Wang F, Mao X, Li Y (2016). *DepR1*, a *TetR* family transcriptional regulator, positively regulates daptomycin production in an industrial producer, *Streptomyces roseosporus* SW0702. Appl Environ Microb.

